# A Prognostic Signature for Colon Adenocarcinoma Patients Based on m6A-Related lncRNAs

**DOI:** 10.1155/2023/7797710

**Published:** 2023-02-13

**Authors:** Su-Zhe Zhou, Ying-Lian Pan, Qing-Chun Deng, Chang-Jun Yin, De-Jiang Zhou, Ming-Liang Liu, Jun Zhou, Xiao-Jing Wu

**Affiliations:** ^1^Department of General Practice, Hefei BOE Hospital, Hefei 230013, China; ^2^Department of Medical Oncology, The First Affiliated Hospital of Hainan Medical University, Haikou 570102, China; ^3^Department of Gynecology, The Second Affiliated Hospital of Hainan Medical University, Haikou 570102, China; ^4^Department of Gastrointestinal Surgery, Sun Yat-Sen Memorial Hospital of Sun Yat-Sen University, Guangzhou 510120, China; ^5^Department of Gastrointestinal Surgery, Affiliated Hospital of Jining Medical College, Jining 272007, China; ^6^Department of Gastrointestinal Surgery, The Third Affiliated Hospital of Guangzhou Medical University, Guangzhou 510150, China; ^7^Department of Surgery Critical Care Medicine, Beijing Shijitan Hospital, Capital Medical University, Beijing 100038, China

## Abstract

N6-methyladenosine (m6A) modification is a common epigenetic modification. It is reported that lncRNA can be regulated by m6A modification. Previous studies have shown that lncRNAs associated with m6A regulation (m6A-lncRNAs) serve as ideal prognostic biomarkers. However, whether lncRNAs are involved in m6A modification in colon adenocarcinoma (COAD) needs further exploration. The objective of this study was to construct an m6A-lncRNAs-based signature for patients with COAD. We obtained the RNA sequencing data and clinical information from The Cancer Genome Atlas (TCGA). Pearson correlation analysis was employed to recognize lncRNAs associated with m6A regulation (m6A-lncRNAs). 24 prognostic m6A-lncRNAs was identified by univariate Cox regression analysis. Gene set enrichment analysis (GSAE) was used to investigate the potential cellular pathways and biological processes. We have also explored the relationship between immune infiltrate levels and m6A-lncRNAs. Then, a predictive signature based on the expression of 13 m6A-lncRNAs was constructed by the Lasso regression algorithm, including UBA6-AS1, AC139149.1, U91328.1, AC138207.5, AC025171.4, AC008760.1, ITGB1-DT, AP001619.1, AL391422.4, AC104532.2, ZEB1-AS1, AC156455.1, and AC104819.3. ROC curves and K M survival curves have shown that the risk score has a well-predictive ability. We also set up a quantitative nomogram on the basis of risk score and prognosis-related clinical characteristics. In summary, we have identified some m6A-lncRNAs that correlated with prognosis and tumor immune microenvironment in COAD. In addition, a potential alternative signature based on the expression of m6A-lncRNAs was provided for the management of COAD patients.

## 1. Introduction

Colon adenocarcinoma is a common pathological type of colon cancer, and its prognosis is poor [1, 2]. Therapies for colon adenocarcinoma (COAD) include surgery, chemotherapy, and radiation therapy [3]. Surgery can cure about half of the COAD patients, but the recurrence rate stays high after surgery. Chemotherapy and radiation are not effective due to their side effects and drug resistance. Besides, the significant heterogeneity of COAD limits the utilization of traditional methods [4]. With the approach of the era of personalized therapy managements, traditional diagnosing failed to satisfy advanced diagnoses and therapies. It is expected to build more useful prognostic signatures to help improve personalized treatment management.

N6-methyladenosine (m6A) modification is one of the principal internal modifications of RNA and participates in many biological processes [5, 6]. m6A modification has been proven to be a reversible process, which is regulated by methyltransferase (writer), demethylase (eraser), and signal sensor (reader) [7]. Studies have reported that m6A modification plays critical roles in the progression of different malignant tumors, including COAD [8–14]. For instance, METTL3 has been identified to promote COAD occurrence and progression by relying on IGF2BP1/IGF2BP2 [15, 16]. It has been found that METTL14 suppresses COAD occurrence and progression by relying on YTHDF2 [17, 18].

lncRNA is a kind of RNA molecule that does not encode a protein, with a length of more than 200 bp, and plays important roles in the carcinogenesis and progression of cancers, including COAD [19, 20]. It has been proven that m6A modification can regulate the physiological functions of lncRNAs [21, 22]. For example, the structure of lncRNA can be regulated by binding to m6A readers, allowing m6A residues to be accessed by specific RNA-binding proteins [23, 24]. m6A modification modulates the structure of lncRNA MALAT1 to play the function of the structural switch, which is related with cancer malignancies [25]. METTL16 (writer) was identified as an RNA-binding protein of lncRNA MALAT1 [26]. m6A modification can stabilize lncRNA FAM225A that served as a sponge for miR-1275 and miR-590-3p in nasopharyngeal carcinoma [27]. METTL3 (writer) can stabilize and upregulate LINC00958, which is involved with the malignancy of liver cancer progression by sponging miR-3619-5p [28]. Previous studies have shown that m6A-lncRNAs serve as ideal prognostic biomarkers. Nevertheless, whether lncRNAs are involved in the regulation of m6A modification in COAD still need to be elucidated. The objective was to identify prognostic m6A-lncRNAs and construct an m6A-lncRNAs-based prognostic signature for patients with COAD.

In this study, we obtained the RNA sequencing data in TCGA and identified 24 prognostic m6A-lncRNAs and 13 m6A-lncRNAs were selected by the Lasso regression algorithm to construct prognostic signature. We have also verified the reliability of the prognostic signature. In addition, a quantitative prognostic nomogram was constructed based on signature and clinical features.

## 2. Methods

### 2.1. Data Source and Preparation

RNA sequencing data of 398 COAD patients with clinical information were obtained from TCGA. 19 COAD patients were excluded due to a lack of necessary clinical information. Subsequently, RNA sequencing data were divided into mRNAs and lncRNAs using the Ensembl Genome Browser [29]. The corresponding clinical data included age, gender, tumor-node-metastasis (TNM) stage, pathological stage, and survival time. We randomly divided COAD patients at a ratio of 7 : 3 into the training cohort (267 patients) and validation cohort (112 patients).

### 2.2. Identifying Prognosis-Related m6A-lncRNAs

23 m6A regulators, based on published articles, including 8 writers, 13 readers, and 2 erasers, were collected in this study (Table 1) [30, 31]. Pearson correlation analysis was applied to the expression of lncRNAs and the 23 m6A regulators to recognize m6A-associated lncRNAs. Univariate Cox regression analysis was applied to recognize prognostic m6A-lncRNAs. We used the “limma” R software package to analyse the differential expression of prognosis-related m6A lncRNA.

### 2.3. Consensus Clustering Analysis

For a better understanding of the role of m6A-lncRNAs, consensus clustering was performed by the “ConsensusClusterPlus” R package to divide all samples into different clusters based on the expression of prognosis-relatedm6A-lncRNAs [32]. Subsequently, Kaplan Meier analysis was performed in different clusters. We also applied the CIBERSORT algorithm and the Wilcoxon test to analyse different immune cell infiltration between clusters. Besides, immune, stromal, and ESTIMATE scores were calculated by the “ESTIMATE” R package. GSEA software was applied to investigate the potential cellular pathways and biological processes in different clusters.

### 2.4. Development and Evaluation of Prognostic Signature

After prognosis-related m6A-lncRNAs were identified, LASSO regression analysis was applied to setup a risk model by “glmnet” R package, which could avoid overfitting by disposing of highly correlated lncRNAs [33, 34]. The signature was calculated in the following format:

(1) $$\begin{array}{c} risk\;score=\overset{n}{\underset{i=1}{\sum }}coef\left(i\right)*lncRNA\left(i\right)\;expression. \end{array}$$

Then, COAD patients were classified into high- and low-risk subgroups. K M survival analysis was employed to compare whether there were differences in survival between the two subgroups using “survival” R packages. To testify the prediction efficacy of the risk model, we employed ROC curves and measured the AUC values by R package “timeROC” [35].

### 2.5. Establishment of Prognostic Nomogram

To further evaluate the reliability of the signature, a comprehensive analysis of risk score and clinical features was performed. Subsequently, a quantitative nomogram was developed on the basis of risk score and clinical features using the “rms” R package [36]. We applied the calibration curves to outline the accuracy of the nomogram.

### 2.6. Statistical Analysis

All statistical analysis in this study was performed using R software (V 4.0.4). The Wilcoxon's test was employed to compare the difference. Kaplan Meier (K M) survival analysis was performed by using the log-rank test. Unless otherwise stated, *P* < 0.05 was considered a statistically significant difference.

## 3. Results

### 3.1. Identification of Prognosis-Related m6A-lncRNAs

**Figure 1:**
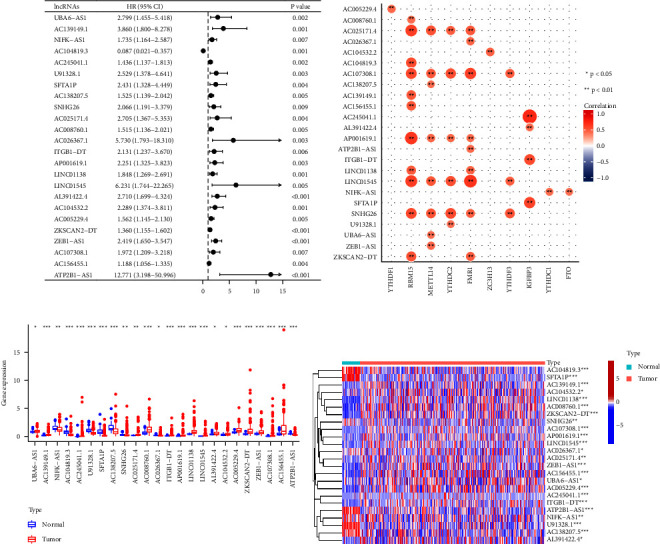
Identification of prognostic m6A-lncRNAs. (a) Forest plots of 24 prognosis-relatedm6A-lncRNAs. (b) The correlation between lncRNAs and m6A regulators. (c) Boxplots of 24 prognosis-related m6A-lncRNAs. (d) Heatmap of 24 prognosis-related m6A-lncRNAs.

By Pearson correlation analysis, a total of 1505 lncRNAs were identified as m6A lncRNA, with an absolute correlation coefficient >0.4 (*P* < 0.001). Then, 24 prognostic m6A-lncRNAs were identified by univariate Cox regression analysis. Among them, UBA6-AS1, AC139149.1, NIFK-AS1, AC245041.1, U91328.1, SFTA1P, AC138207.5, SNHG26, AC025171.4, AC008760.1, AC026367.1, ITGB1-DT, AP001619.1, LINC01138, LINC01545, AL391422.4, AC104532.2, AC005229.4, ZKSCAN2-DT, ZEB1-AS1, AC107308.1, AC156455.1, and ATP2B1-AS1 were recognized as risky lncRNAs for HR > 1 (*P*  < 0.05) and AC104819.3 was recognized as protective lncRNA with HR < 1 (*P* < 0.05) (Figure 1(a)). The correlation between lncRNAs and m6A regulators is shown in Figure 1(b). The expression of 24 prognostic m6A-lncRNAs in normal tissues and tumor tissues was displayed by the box plot and heatmap. (Figures 1(c) and 1(d)).

### 3.2. Consensus Clustering Analysis

**Figure 2:**
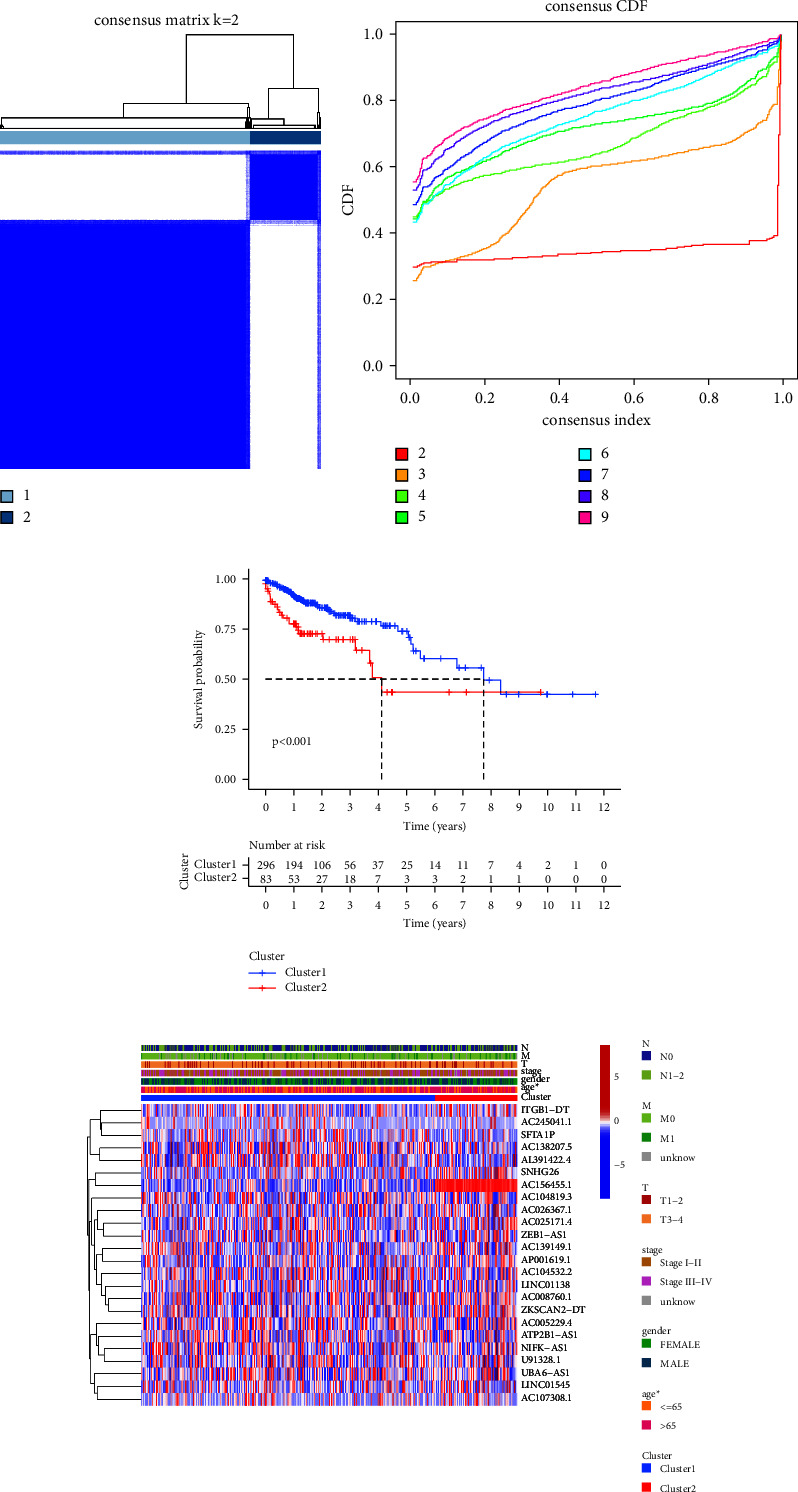
Consensus cluster classification. (a) Consensus clustering matrix when *k* = 2. (b) The cumulative distribution functions (CDFs) plot for *k* = 2–9. (c) The K M survival curves for COAD patients in 2 clusters. (d) Heatmap of 24 prognosis-related m6A-lncRNAs expression along with clinicopathological features in 2 subgroups. *Note*. ^*∗*^*P* < 0.05.

For further understanding the roles of prognostic m6A-lncRNAs, patients were clustered according to the expression of m6A-lncRNAs by consensus clustering analysis. As displayed in the consensus matrix map and cumulative distribution function (CDF) plot for *k* = 2, the interference was the smallest (Figures 2(a) and 2(b)). The overall survival results of patients in cluster 1 are better than those in cluster 2 (Figure 2(c)).  Figure 2(d) displays the correlation between clinicopathological features and clusters.

### 3.3. Gene Set Enrichment Analysis (GSAE)

**Figure 3:**
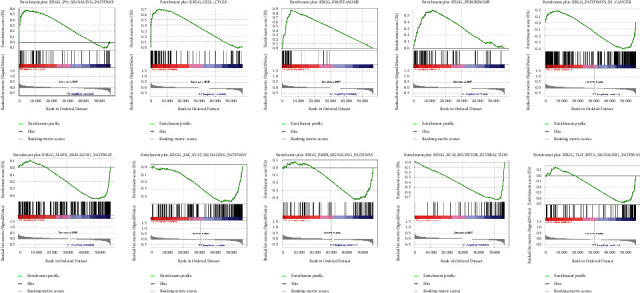
Gene set enrichment analysis.

To explore the potential cellular pathways and biological processes of prognostic m6A-lncRNAs, the GSEA was employed between two clusters. As displayed in Figure 3, genes in cluster 2 were enriched in the p53-signaling-pathway, proteasome, cell cycle, and peroxisome. Besides, genes in cluster 1 were enriched in TGF-betasignaling-pathway, ERBB-signaling-pathway, ECM-receptor interaction, MAPK-signaling-pathway, and JAK-STAT-signaling-pathway.

### 3.4. Immune Cell Infiltration and Distribution of Immunity

**Figure 4:**
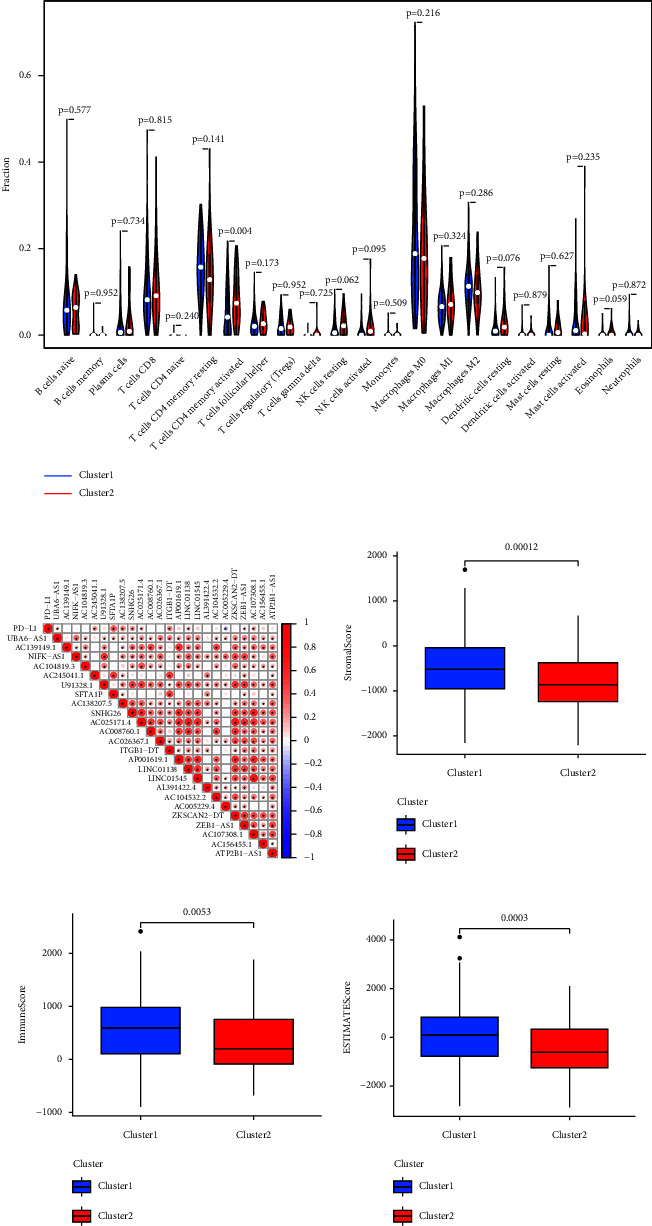
Immune cell infiltration and the distribution of immunity. (a) Infiltrating levels of 22 types of immune cells. (b) Correlation between the m6A-lncRNAs and PD-L1. (c–e) Stromal score, immune score, and ESTIMATE score in two clusters.

To explore the roles of m6A-lncRNAs in the tumor immune microenvironment (TIME), we compared the scores of 22 different immune cell types in two clusters. As shown in Figure 4(a), activated memory CD4 T cells are rich in cluster 2 (*P* < 0.05). Figure 4(b) shows the positive correlation between the m6A-lncRNAs and PD-L1. Furthermore, we analysed the distribution of immunity in two clusters. Cluster 1 have a higher stromal score, immune score, and ESTIMATE score (*P* < 0.05, Figures 4(c)–4(e)).

### 3.5. Construction and Validation of m6A-lncRNAs Signature

**Figure 5:**
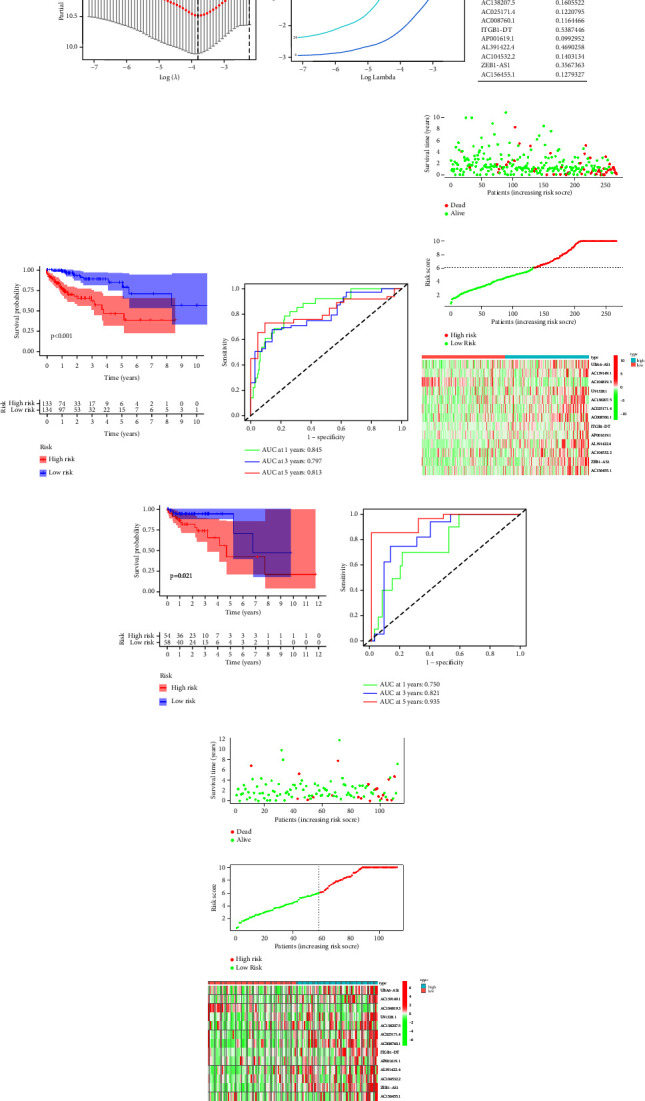
Construction and validation of prognostic signature. (a, b) Calculation of the optimal *λ* in Lasso regression analysis. (c) 13 prognosis lncRNAs and each of its coefficients. (d, e) The K M survival curves and ROC curves in training cohort. (g, h) The K M survival curves and ROC curves in validation cohort. (f, i) Survival time scatter plots and risk score distribution plots in training set and internal validation set.

The LASSO regression algorithm was used to avoid overfitting and for constructing risk scores. As displayed in Figures 5(a) and 5(b), at penalty factor (*λ*) 13, the coefficients of some variables are near to 0 [37]. Ultimately, 13 m6A-lncRNAs were identified as independent prognostic factors. The m6A-lncRNAs risk model for predicting prognosis in COAD was established on the basis of coefficients of 13 m6A-lncRNAs in the following format: risk score = UBA6-AS1 *∗*∗ 0.7684476 + AC139149.1 *∗* 0.7685387 + AC104819.3 *∗* (−1.96469) + U91328.1 *∗* 0.3846686 + AC138207.5 *∗* 0.1605522 + AC025171.4 *∗* 0.1220795 + AC008760.1 *∗* 0.1164466 + ITGB1-DT *∗* 0.5387446 + AP001619.1 *∗* 0.0992952 + AL391422.4 *∗* 0.4690258 + AC104532.2 *∗* 0.1403134 + ZEB1-AS1 *∗* 0.3567363 + AC156455.1 *∗* 0.1279327 (Figure 5(c)).

The K M curves showed that the survival outcome of the low-risk subgroup was better (*P* < 0.05) (Figures 5(d) and 5(g)). The time-dependent ROC curves indicated that, in the training cohort, the AUC value at 1 year was 0.845, 0.797 at 3 years, and 0.813 at 5 years (Figure 5(e)). In the validation cohort, the AUC value at 1 year was 0.750, 0.821 at 3 years, and 0.935 at 5 years (Figure 5(h)). Furthermore, the risk score distribution plot and scatter plot also showed that the survival outcome of the high-risk subgroup was worse. The heatmap illustrated that the expressions of risky lncRNAs were upregulated in the high-risk group, while protective lncRNA was downregulated (Figures 5(f) and 5(i)).

**Figure 6:**
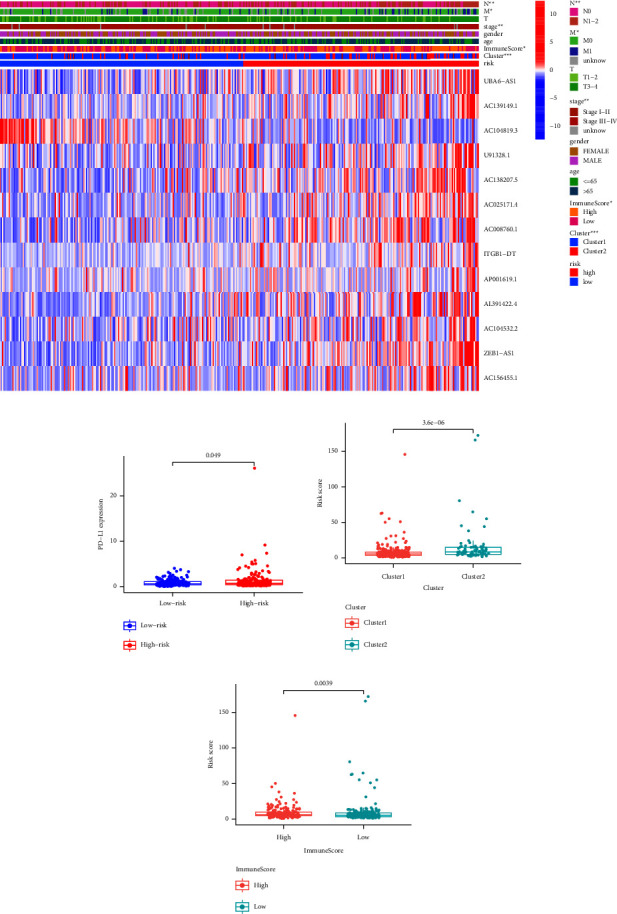
The correlation of risk score and clinical characteristics. (a) Heatmap of m6A-lncRNAs expression, immune score, and clinical features in low- and high-risk score subgroup. (b) Distribution of PD-L1 expression stratified by risk scores. (c, d) Distribution of risk scores stratified by clusters and immune score.

To further validate the risk model, the correlation between risk score and clinical characteristics was analysed. As shown in Figure 6(a), the risk score was significantly correlated with pathological stage and immune score (*P* < 0.05). Besides, the scatter plot showed that the risk score was also significantly correlated with PD-L1 expression and immune score (Figures 6(b) and 6(d)).

### 3.6. Development of Survival Prognostic Nomogram

**Figure 7:**
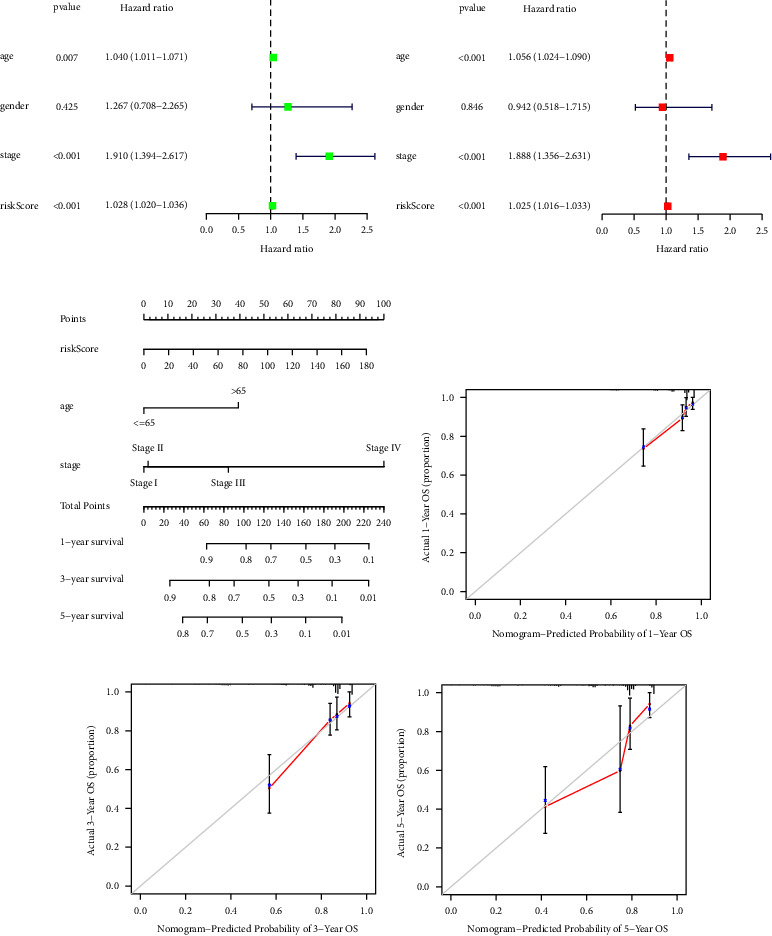
Construction of survival prediction nomogram. (a, b) The univariate and multivariate Cox regression analysis. (c) Nomogram based on risk score and prognostic clinical characteristics. (d–f) The calibration curves of the prognostic nomogram.

We comprehensively analysed the risk score and clinical characteristics. Risk scores, age, and pathological stage were identified as significant independent prognostic variables (*P* < 0.05, Figures 7(a) and 7(b)), which revealed that the risk model served as a reliable tool for COAD patients. Subsequently, we constructed a quantitative nomogram on the basis of risk score and clinical characteristics (Figure 7(c)). The calibration curves displayed the concordance between observed and predicted overall survival (Figures 7(d)–7(f)).

## 4. Discussions

N6-methyladenosine (m6A) modification is a common epigenetic modification in eukaryotes, involving many biological processes, such as RNA splicing, translation, and expression. M6A modification and lncRNAs play critical roles in the biological processes of COAD. For example, m6A regulators, such as METTL3, METTL14, and YTHDF2, have been proven to be involved in regulating the pathological process of COAD [16, 17]. It is reported that lncRNA can be regulated by m6A modification. However, whether lncRNAs are involved in the regulation of m6A modification in the progression of COAD needs further exploration.

With the approach of the era of personalized therapy managements, traditional diagnosing failed to satisfy advanced diagnoses and therapies. It is expected to build more useful prognostic signatures to help improve personalized treatment management. In this study, we focused on identifying prognosis-related lncRNAs associated with m6A modification (m6A-lncRNAs) and used bioinformatics methods to establish a reliable risk model for patients with COAD.

We downloaded RNA sequencing data in TCGA. M6A-lncRNAs were recognized by Pearson correlation analysis according to the expression of lncRNAs and m6A regulators. There are 24 prognostic m6A-lncRNAs identified by univariate Cox regression analysis. For exploring the biological features of these 24 prognostic m6A-lncRNAs, we divided the COAD patients into two clusters. GSAE has been applied to investigate the potential cellular pathways and biological processes. Accumulated studies have illustrated that tumor immune microenvironment (TIME) is correlated with tumorigenesis and the development of COAD [38–41]. In this study, we have explored the relationship between immune infiltrate levels and m6A-lncRNAs. There are also significant differences between the two clusters in ESTIMATE score, stromal score, and immune Score.

The Lasso regression algorithm is widely used to construct risk models. The most important difference between lasso regression analysis and traditional stepwise Cox regression analysis is that it can process all variables simultaneously, instead of step by step [34], which greatly improves the stability of the model. In this study, LASSO regression analysis was conducted to avoid overfitting and to construct risk scores. Ultimately, 13 m6A-lncRNAs, including UBA6-AS1, AC139149.1, U91328.1, AC138207.5, AC025171.4, AC008760.1, ITGB1-DT, AP001619.1, AL391422.4, AC104532.2, ZEB1-AS1, AC156455.1, and AC104819.3 were selected to construct the signature. To testify the reliability of the signature, COAD patients were divided, at a ratio of 7 : 3, into a training set and validation set. K M survival analysis and the ROC curves showed well discrimination of the signatures. In addition, risk score and clinicopathological characteristics were integrated into the analysis. Age, risk score, and pathological stage were recognized as independent prognostic factors, which also demonstrated the reliability of the risk model. Then, a quantitative prognostic nomogram was constructed. The calibration curves demonstrated the accuracy of the nomogram.

There were also limitations in our study. First, the m6A-lncRNAs risk model was constructed and validated based on the TCGA database. We did not verify the prognostic signatures in external independent cohorts. Second, the interaction between prognosis-related m6A-lncRNA and m6A regulator lacks experimental confirmation.

## 5. Conclusions

To sum up, we have identified some m6A-related lncRNAs which were correlated with prognosis and tumor immune microenvironment. A reliable alternative prognostic signature was provided for the management of COAD patients. We also combined risk scores with clinical characteristics to establish a quantitative prognostic nomogram.

## Figures and Tables

**Table 1:** N6-methyladenosine (m6A) modification regulators.

Eraser	FTO; ALKBH5
Writer	ZC3H13; METTL16; METTL14; METTL3; VIRMA; RBM15B; RBM15; WTAP
Reader	FMR1; YTHDF3; YTHDF2; YTHDF1; YTHDC2; YTHDC1; RBMX; HNRNPC; HNRNPA2B1; LRPPRC; IGFBP3; IGFBP2; IGFBP1

## Data Availability

The data analysed in this study are free available in The Cancer Genome Atlas (TCGA) database (https://portal.gdc.cancer.gov).
